# PRMT5 Inhibition Promotes PD-L1 Expression and Immuno-Resistance in Lung Cancer

**DOI:** 10.3389/fimmu.2021.722188

**Published:** 2022-01-17

**Authors:** Rui Hu, Bingqian Zhou, Zheyi Chen, Shiyu Chen, Ningdai Chen, Lisong Shen, Haibo Xiao, Yingxia Zheng

**Affiliations:** ^1^ Department of Thoracic Surgery, Xin Hua Hospital, Shanghai Jiao Tong University School of Medicine, Shanghai, China; ^2^ Department of Laboratory Medicine, Xin Hua Hospital, Shanghai Jiao Tong University School of Medicine, Shanghai, China; ^3^ Faculty of Medical Laboratory Science, Shanghai Jiao Tong University School of Medicine, Shanghai, China; ^4^ Xin Hua Children’s Hospital, Shanghai Jiao Tong University School of Medicine, Shanghai, China

**Keywords:** PD-L1, GSK591, lung cancer, PRMT5, immuno-resistance

## Abstract

Protein arginine transferase 5 (PRMT5) has been implicated as an important modulator of tumorigenesis as it promotes tumor cell proliferation, invasion, and metastasis. Studies have largely focused on PRMT5 regulating intrinsic changes in tumors; however, the effects of PRMT5 on the tumor microenvironment and particularly immune cells are largely unknown. Here we found that targeting PRMT5 by genetic or pharmacological inhibition reduced lung tumor progression in immunocompromised mice; however, the effects were weakened in immunocompetent mice. PRMT5 inhibition not only decreased tumor cell survival but also increased the tumor cell expression of *CD274 in vitro* and *in vivo*, which activated the PD1/PD-L1 axis and eliminated CD8+T cell antitumor immunity. Mechanistically, PRMT5 regulated *CD274* gene expression through symmetric dimethylation of histone H4R3, increased deposition of H3R4me2s on *CD274* promoter loci, and inhibition of *CD274* gene expression. Targeting PRMT5 reduced this inhibitory effect and promoted *CD274* expression in lung cancer. However, PRMT5 inhibitors represent a double-edged sword as they may selectively kill cancer cells but may also disrupt the antitumor immune response. The combination of PRMT5 inhibition and ani-PD-L1 therapy resulted in an increase in the number and enhanced the function of tumor-infiltrating T cells. Our findings address an unmet clinical need in which combining PRMT5 inhibition with anti-PD-L1 therapy could be a promising strategy for lung cancer treatment.

## Introduction

Protein arginine methylation is a common posttranslational modification that affects the function of many histones and non-histone proteins and regulates important cellular processes (1). Arginine methylation is mediated by protein arginine methyltransferases (PRMTs), which utilize S-adenosyl methionine as a methyl donor to catalyze mono- and dimethylation of the guanidino group of arginine residues (2). PRMT5 is a type II protein arginine methyltransferase that dimethylates histones, such as H3, H4, and H2A, and non-histone proteins, such as p53, p65, and HOXA9, to regulate various cellular processes including mRNA splicing, DNA repair, signal transduction, cell cycle, apoptosis, and oncogenesis (3, 4). Histone posttranslational methylation leads to changes in chromatin composition and configuration, is a principal component of epigenetic-mediated gene expression, and induces transcriptional activation or repression (5). PRMT5 drives or represses gene expression by modifying histone residues. It dimethylates histone H3R2me2s, drives H3K4me3, and promotes gene expression, whereas it dimethylates H3R8 and H4R3 to repress gene activation (4, 6).

Several studies have demonstrated high expression of PRMT5 in various tumor types including lung cancer, breast cancer, hematologic malignancies, glioblastoma, and colorectal cancer, and these studies demonstrate an important role for PRMT5 in tumorigenesis (7–11). Thus, PRMT5 is an attractive cancer target for small-molecule inhibition. Targeting PRMT5 is currently being evaluated in clinical trials in both hematologic and solid tumors, including primary and metastatic tumors, to determine efficacy and toxicity in patients (12).

Several studies have demonstrated that PRMT5 deletion affects immune cell development. Deletion of PRMT5 in all cell types is disruptive to embryonic development, and T-cell-specific deletion of PRMT5 causes a decrease in the number of thymus iNK cells and peripheral CD4+ and CD8+ T cells (13, 14). B cell-specific deletion of PRMT5 leads to reduced germinal center formation and causes B cell apoptosis (15). Inhibition of PRMT5 promotes CD8+ T cell apoptosis by upregulating P53 expression and reducing AKT pathway activity (16). PRMT5 promotes cholesterol biosynthesis and mediates Th17 responses in experimental autoimmune encephalomyelitis (EAE). Selective inhibition of PRMT5 reduces the clinical score of EAE (17, 18). These studies indicate that PRMT5 plays an important role in regulating immune cell development, including T cells, B cells, and iNKT cells.

Lung cancer is one of the most common types of malignancies worldwide and is associated with a high mortality rate. It has been the subject of extensive studies with respect to pathogenesis, diagnosis, treatment, and prognosis. Recently, immunotherapies targeting checkpoints have been evaluated in lung cancer in addition to traditional surgical techniques, radiotherapy, and chemotherapy. PD-1 expression on tumor-infiltrating lymphocytes interacts with PD-L1 expressed on tumors and/or immune cells in the tumor microenvironment (TME), thus attenuating effector T cell responses and enabling tumors to escape immune attack. Anti-PD1 mAb (nivolumab) and anti-PD-L1 mAb (atezolizumab) have been approved for lung cancer treatment; however, the overall response rate is limited (19). Exploring innovative therapies, such as targeting oncogenesis factors, with checkpoint inhibitors may represent a promising strategy to treat lung cancer.

PRMT5 expression is elevated in lung cancer, and the TGFβ–PRMT5–MEP50 axis regulates cancer cell invasion through histone H3 and H4 arginine methylation, which couples transcriptional activation and repression (7). This suggests that PRMT5 is a promising target for lung cancer treatment; however, the pharmacology of PRMT5 inhibition and its effects on immune cells are largely unknown. In the present study, we implanted syngeneic Lewis lung carcinoma (LLC) cells subcutaneously into normal and immunodeficient mice and treated them with the PRMT5-specific inhibitor, GSK591. We found that targeting PRMT5 was more effective on immunodeficient mice compared with immunocompetent mice. Moreover, we found that GSK591 treatment not only inhibited tumor cell survival but also induced tumor cell expression of type 1 IFN response and *CD274* genes, which activated the PD1/PD-L1 axis and eliminated T cell antitumor activity. Mechanistically, PRMT5 regulated *CD274* gene expression through symmetric dimethylation of histone H4R3 and high level of H3R4me2s deposition on the *CD274* promoter loci and repressed *CD274* expression, which resulted in the inhibition of PRMT5-induced *CD274* expression. Overall, our results demonstrate that PRMT5 inhibition alone inhibited lung cancer progression but induced PD-L1 expression that compromised the antitumor activity of CD8+ T cells. Combining PRMT5 inhibition with anti-PD-L1 therapy synergistically inhibited the growth of lung cancer cells and activated CD8+T cell immune surveillance, which may be an effective approach for lung cancer treatment.

## Materials and Methods

### Cells and Clinical Samples

Lung cancer cell lines, NCI-H460, HCC827, and LLC, were purchased from the Chinese Academy of Sciences Cell Bank. All cells were cultured at 37°C in a humidified incubator with 5% CO_2_ in Dulbecco’s modified Eagle’s medium (DMEM) (HyClone) or Roswell Park Memorial Institute (RPMI) 1640 medium (Gibco) containing 10% fetal bovine serum (FBS) (Gibco), 100 U/ml penicillin, and 100 μg/ml streptomycin (Gibco). For PRMT5 inhibition, 1 × 10^5^ cells were seeded into 24-well plates. GSK591 (Selleck) was diluted in DMSO and added to each culture at final concentrations of 250 nM or 1 μM, and the cells were harvested for further analysis.

### Animal Experiments

Male C57BL/6 and BALB/C nude mice were purchased from the Shanghai Laboratory Animal Center, Chinese Academy of Sciences (Shanghai, China). The animals were housed in the animal care facility of Shanghai Jiao Tong University School of Medicine, Xin Hua Hospital, under pathogen-free conditions. This study was carried out in accordance with the recommendations of the Institutional Animal Care and Use guidelines, Xin Hua Hospital Committee. The protocol was approved by the Institutional Animal Care and Use Committee of Xin Hua Hospital.

For subcutaneous tumorigenicity experiments. LLC cells (1.5 × 10^6^ in 150 μl, 50% Matrigel) were subcutaneously implanted into the right flanks of the nude mice. GSK591 treatment was initiated when the tumor size reached 100 mm^3^ (9 days after inoculation). Mice were randomly assigned into two groups. Animals in the GSK591 or vehicle (5% DMSO + 30% PEG300 + 65% water) groups were injected intraperitoneally at a dose of 50 mg/kg for 12 days. For the *in vivo* blockade experiments, LLC cells (1.5 × 10^6^) were injected subcutaneously into 6-week-old C57BL/6 mice. Nine days after tumor inoculation, the mice were randomly divided into four groups (IgG + vehicle, IgG + GSK591, anti-PD-L1 + vehicle, and anti-PD-L1 + GSK591). Mice were injected intraperitoneally with GSK591 (50 mg/kg) for 12 days. The mice were injected intraperitoneally with anti-PD-L1 mAb or mouse IgG control (50 mg/kg) once every 3 days (days 9, 12, 15, and 18).

### Isolation and Culture of CD8 T Cells

CD8 T cells (human) were obtained from peripheral blood mononuclear cells (PBMCs) by magnetic cell separation using the human CD8^+^T Cell Isolation Kit from Miltenyi Biotec and stimulated with 2 μg/ml anti-CD3 antibody and 1 μg/ml anti-CD28 antibody (eBioscience). Healthy individuals (n = 20, range 20–58 years) were recruited from Xinhua Hospital, Shanghai Jiaotong University School of Medicine. Prior to participation, written informed consent was obtained from all subjects. All studies were performed in accordance with the Declaration of Helsinki. The study was approved by the Research Ethics Board of Xinhua Hospital, Shanghai Jiao Tong University School of Medicine.

CD8 T cells (mouse) were generated from murine spleens by magnetic separation using the EasySep Mouse CD8 T Cell Isolation Kit from STEMCELL. Freshly isolated CD8 T cells were activated with 2 μg/ml anti-CD3 antibody and 1 μg/ml anti-CD28 antibody in RPMI-1640 with FBS. For PRMT5 inhibition, the cells were treated with 250 nM and 1 μM GSK591.

### Cell Transfection

Human PRMT5 knockdown was achieved using the short hairpin RNA (shRNA)-mediated stable silencing method. Three sequences of short hairpin RNAs targeting PRMT5 were designed as follows: shRNA1, GCC CAG TTT GAG ATG CCT TAT; shRNA2, TTC GGC TCA AGC CAC CAA TCT ATG; shRNA3, CCC ATC CTC TTC CCT ATT AAG; and control shRNA, TTC TCC GAA CGT GTC ACG T. 293T cells were seeded at 5 × 10^4^ cells per well in 24-well plates. Cell transfection was performed using Lipofectamine 2000 (Invitrogen). The virus was collected after 48 h of transfection and used to infect NCI-H460 cells. PRMT5 stable knockdown cells were obtained after selecting with 2 μg/ml puromycin. For murine PRMT5 knockdown, three sequences of short hairpin RNA targeting PRMT5 were designed as follows: shRNA1, GCG GCG ATG GCA GTC GGA GGT GCT GGT GG; shRNA2, AGC CAG GTG ACA GTT GTC TCA TCA GAC AT; and shRNA3, TTC CTG TGG AGG TGA ACA CGG TGC TTC AT. Cell transfection was performed using Lipofectamine 2000 (Invitrogen).

### Cell Proliferation

Cells were seeded into a 96-well plate at 2 × 10^3^ cells per well with five duplicates. Next, 10% Cell Counting Kit-8 (CCK-8, Dojindo Laboratories, Japan) was added to the culture medium and incubated for 2 h at 37°C. Cell viability was monitored by measuring the absorbance at 450 nm using a PowerWave XS microplate reader (BioTek, Winooski, VT, USA).

### *In Vitro* Cell Migration Assay

Cells were suspended in serum-free DMEM and seeded into transwell inserts containing an 8-μm microporous filter (Corning, USA). The lower compartment contained DMEM with 10% FBS to attract the cells. After a 12-h incubation, cells remaining in the upper chamber were removed with flat-bottomed cotton swabs. Cells on the lower surface of the chamber were fixed with 4% paraformaldehyde and stained with 0.5% crystal violet. At least six random microscopic fields (magnification, ×200) were photographed, and the cells were counted.

### Immunohistochemistry Analysis

Immunohistochemistry (IHC) was performed on 4-µm paraffin sections. The slides were placed in a staining module for deparaffinization, epitope retrieval, and endogenous peroxidase quenching. They were then incubated with the primary antibody, anti-PD-L1 (1:100 dilution), for 1 h at room temperature. Detection of primary antibody was done with anti-rabbit HRP, and the reaction was visualized with DAB substrate (Dako REAL EnVision Kit, K5007), whereas hematoxylin was used as a counterstain. Each sample was assigned a score according to the intensity of the staining (0 = no staining, 1 = weak staining, 2 = moderate staining, and 3 = strong staining), and the proportion of stained cells (1 = 1%–25%, 2 = 25%–50%, 3 = 50%–75%, 4 = 75%–100%) was determined. The final score represents the product of the two scores.

### Flow Cytometry

For cell surface marker staining, the following antibodies were used: anti-CD45, anti-CD4, anti-CD3, anti-CD8, anti-PD1, and anti-PD-L1. For intracellular cytokine staining, cells were stimulated with cell stimulation cocktail plus protein transport inhibitors (eBioscience) for 4 h. The cells were fixed and permeabilized with Cytofix/Cytoperm buffer and stained with antibodies against intracellular cytokines and granzyme B. Flow cytometric analysis was performed with a FACSCanto II instrument (BD Bioscience) and FlowJo software (Treestar). Antibody information is provided in **Supplementary Table 1**.

### Quantitative Real-Time PCR

Total RNA was isolated from cells or tissues using TRIzol (Takara). First-strand cDNA was synthesized from total RNA using the RT Master Mix (Takara). Transcript levels were detected using SYBR Green-based real-time PCR and the ABI StepOne qPCR Detection System (Life Technologies). The mRNA levels were normalized to that of β-actin mRNA. Primer sequences are listed in **Supplementary Table 2**.

### Western Blot Analysis

Cells were harvested and lysed in 1× SDS loading buffer (Beyotime), electrophoresed on SDS-PAGE gels, and transferred to nitrocellulose membranes (Millipore). The membranes were blocked in PBST containing 5% non-fat milk and incubated with primary antibody overnight at 4°C on a rotator. The following primary antibodies were used: PRMT5 (Abcam), PD-L1 (CST), STAT1 (CST), sDMR (Abcam), and actin (ABclonal). The membranes were incubated with secondary antibodies (Abcam), and the protein signals were detected with a LI-COR Odyssey 499 Imager. The captured images were quantitated using Image Studio Version 3.0 software.

### Chromatin Immunoprecipitation Assay

NCI-H460 and LLC cells were harvested and cross-linked with 1% formaldehyde for 10 min at room temperature. The cells were mixed with glycine and incubated for 5 min at room temperature. Next, the cells were washed with ice-cold PBS and centrifuged. The supernatant was discarded, and the cells were resuspended in lysis buffer supplemented with 5 μl PIC and 5 μl PMSF. The samples were sonicated, and 50 μl of the product was removed to assess DNA fragment size. The remainder was stored at −80°C. Antibodies for control IgG (Abcam) and H4R3 me2s (Active Motif) were used for chromatin immunoprecipitation (ChIP) assay. DNA extracted from 10 μl of pre-immunoprecipitated samples was used as an input control. Beads were washed with ChIP buffer I once and then ChIP buffer II twice. The immunoprecipitated chromatin was eluted with ChIP elution buffer and incubated at 37°C for 15 min, followed by 15 min at 95°C in a thermocycler to reverse the cross-links. Then, 2 μl of Proteinase K was added and incubated at 37°C for 60 min followed by the addition of 2 μl of Proteinase K stop solution. The bound DNA fragments were verified by quantitative real-time PCR (qPCR). The PCR primer sequences for ChIP are shown in **Supplementary Table 2**.

### *In Vitro* Tumor and CD8 T Cell Coculture

Lung cancer cells were counted and plated at a concentration of 10,000 cells/well in a flat 96-well plate. Then, the corresponding activated CD8 T cells were added and cultured with targeted cells at a 10:1 ratio for 24 or 48 h. For antibody blocking experiments, cells were treated with antibody against human PD-L1 or IgG at a final concentration of 5 μg/ml.

### RNA-Seq Analysis

Total RNA was extracted using the RNeasy Plus Mini Kit (Qiagen) according to the manufacturer’s protocol. RNA-seq libraries were prepared using the Illumina TruSeq Stranded mRNA LT Sample Preparation Kit (Illumina). Sequencing was performed on an Illumina HiSeq 2500 set to 50-bp lengths. For RNA analysis, raw reads were mapped to the human reference genome (GRCm38) by Hisat2, and differential expression analysis was done using StringTie (v1.3.4) and Ballgown51. Gene ontology analysis was performed with the Panther Classification System (http://pantherdb.org/).

### Statistical Analysis

All data are presented as the mean ± SEM. Statistical analyses were performed with Prism 8 (GraphPad Software). Data were evaluated using a two-tailed Student’s t test. *p* < 0.05 was considered statistically significant.

## Results

### Specific Knockdown of PRMT5 in the Human Lung Cancer Cell Line Is Associated With Negative Regulation of T Cells

**Figure 1:**
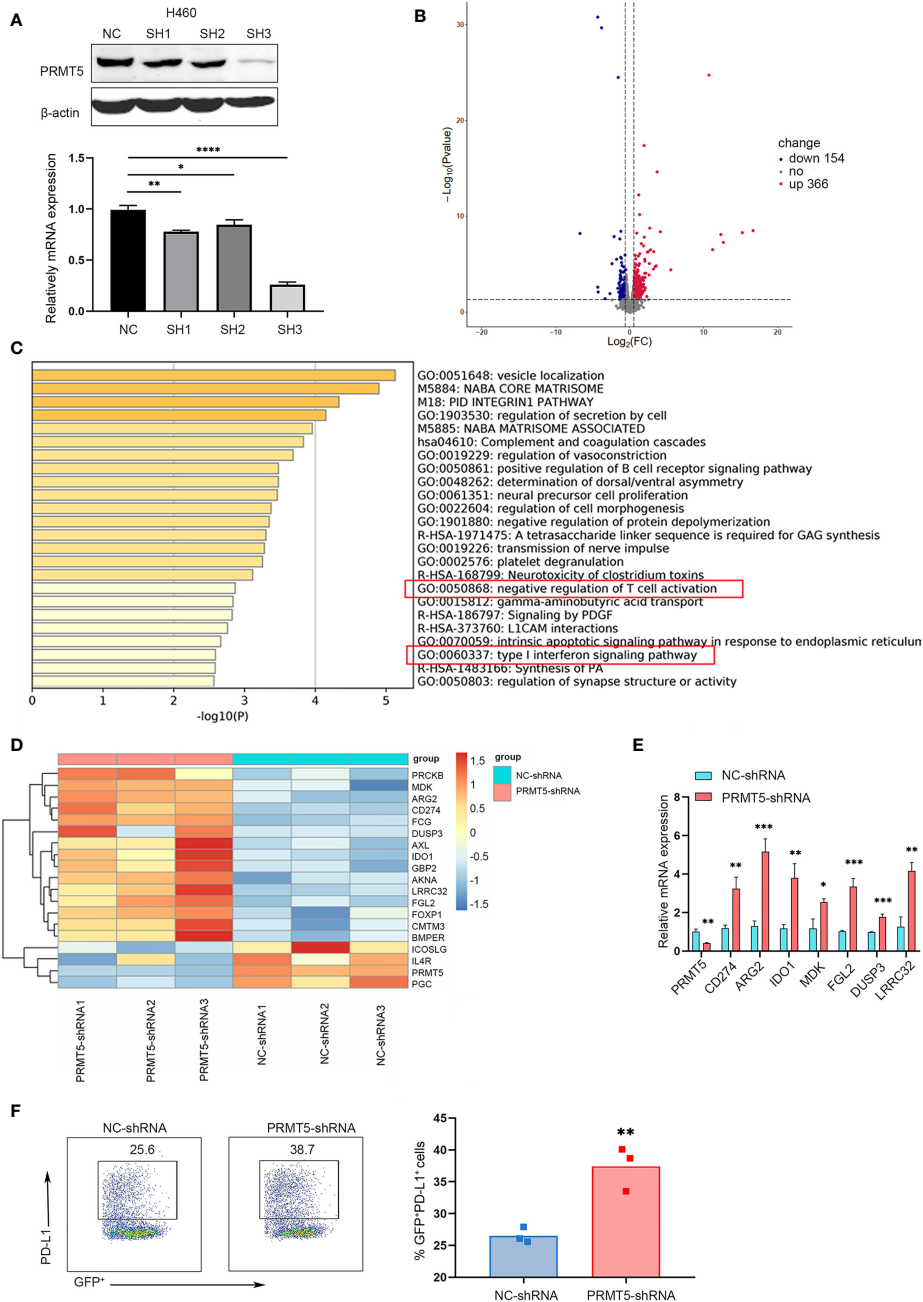
Specific knockdown of PRMT5 in the human lung cancer cell line is related to negative regulation of T cell activation. **(A)** Three shRNA sequences targeting PRMT5 were designed. *PRMT5*-specific or control shRNA was transfected into the NCI-H460 human lung cancer line, and PRMT5 expression was measured by Western blot analysis (top) and qPCR (bottom). Data are the means ± SEM of three independent experiments. Statistical differences were determined by two-tailed unpaired Student’s t test, *p < 0.05, **p < 0.01, ***p < 0.001, ****p < 0.0001. **(B)** Volcano plot showing the up- and downregulated genes in NCI-H460 cells between NC-shRNA and PRMT5-shRNA. Each group contained three replicates. **(C)** Bar graph of enriched terms of the differentially expressed genes between NC-shRNA and PRMT5-shRNA in HCI-H460 cells. Red boxes show the prominent GO terms. **(D)** Heatmap plot showing the negative regulation of T cell activation-associated genes between NCI-H460 PRMT5-shRNA and NC-shRNA cells. **(E)** Quantitative PCR verifying the candidate genes in NCI-H460 PRMT5-shRNA and NC-shRNA cells. The mean ± SEM is graphed. Statistical differences were determined by a two-tailed unpaired Student’s t test, *p < 0.05, **p < 0.01, ***p < 0.001. **(F)** PD-L1 expression was measured in NC-shRNA and PRMT5-shRNA NCI-H460 cells by flow cytometry. Data are representative of three independent experiments. Mean ± SEM was graphed. Statistical differences were determined by a two-tailed unpaired Student’s t test, **p < 0.01.

We verified PRMT5 expression in lung cancer and found that it was increased in lung cancer cell lines compared with the embryonic fibroblast cell line, MRC-5 (**Supplementary Figure 1A**). Next, we used TCGA RNA-seq data of human lung adenocarcinoma and found that PRMT5 expression was much higher in lung tumor tissues compared with normal tissues (**Supplementary Figure 1B**), suggesting that PRMT5 may be involved in tumorigenesis in lung cancer. We constructed a stable system to downregulate PRMT5 in NCI-H460 human lung cancer lines to determine the oncogenic properties of PRMT5. The results indicated that the shRNA sequence targeting the third site of PRMT5 was most effect to knock down PRMT5 expression (**Figure 1A**). Proliferation and migration ability was decreased when PRMT5 expression was reduced (**Supplementary Figures 1C, D**). Moreover, we knocked down PRMT5 expression in another human lung cancer line, HCC827, and the LLC mouse lung cancer cell line to further verify the function of PRMT5. The data showed that inhibition of PRMT5 expression decreased both HCC827 and LLC proliferation and migration ability (**Supplementary Figures 1E-J**). To further explore the role of PRMT5 in lung cancer, we performed RNA sequence analysis on NCI-H460 cells that were stably transfected with PRMT5 shRNA and control shRNA using three biological replicates. The data showed that PRMT5 knockdown cells exhibited different transcriptional profiles compared with control cells (**Figure 1B**). Interestingly, PRMT5 knockdown cells were enriched in the negative regulation of T cells and type I INFγ response genes (**Figure 1C**). We found that negative regulation of T cell molecules, such as Arg2, CD274, and Ido1, was increased in the PRMT5 shRNA group compared with the control group (**Figure 1D**). We verified these molecules by real-time qPCR, and the data were consistent with that of RNA sequencing (**Figure 1E**). Also, flow cytometry data showed that PD-L1 expression was increased in the PRMT5 knockdown cells compared with control cells (**Figure 1F**). These results indicate that although PRMT5 knockdown reduced lung cancer cell proliferation and migration, it may also destroy the antitumor activity of T cells.

### Treatment With the PRMT5 Inhibitor, GSK591, Promotes PD-L1 Expression *In Vitro and In Vivo*

**Figure 2:**
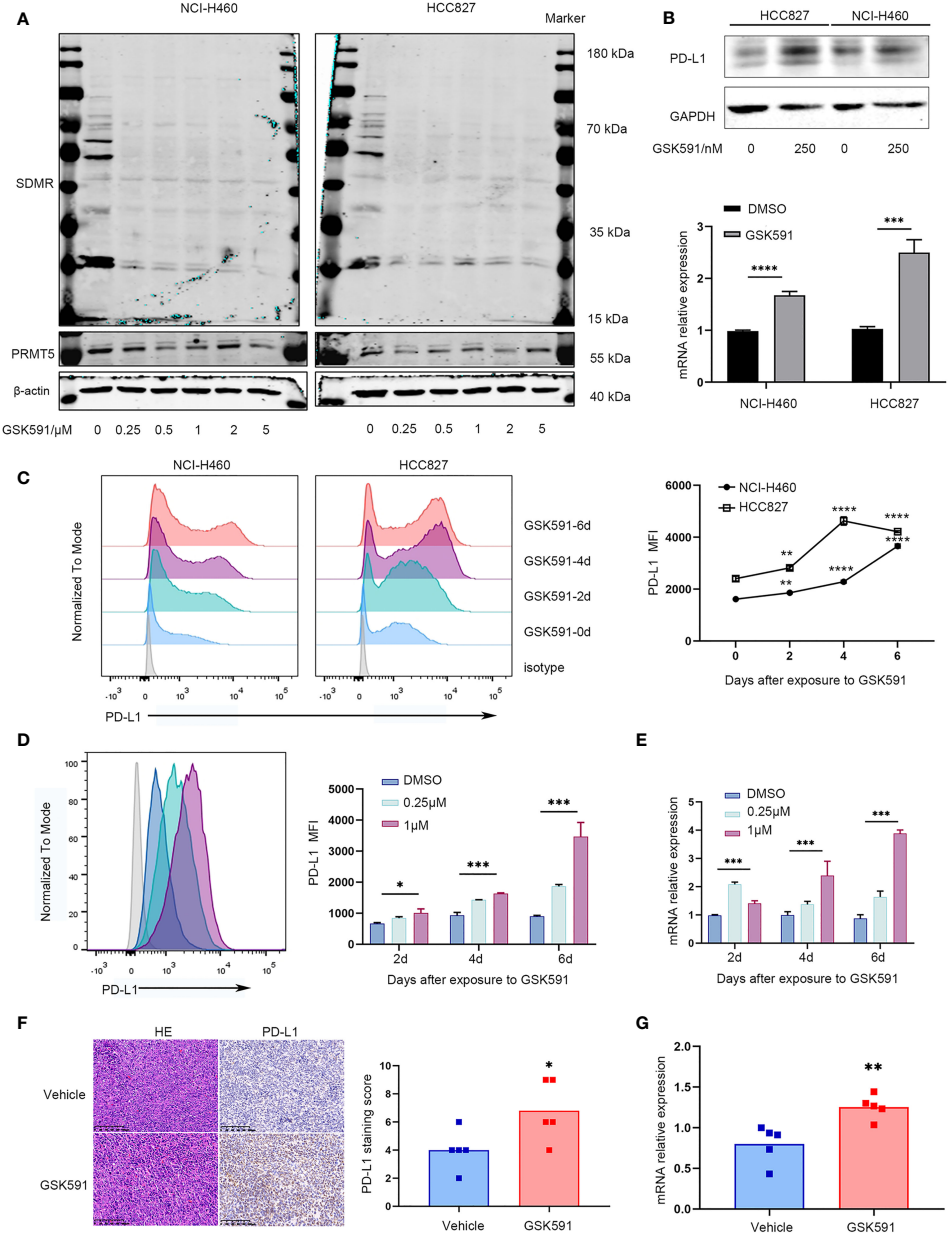
PRMT5 inhibition leads to upregulation of PD-L1 *in vitro* and *in vivo.* **(A)** NCI-H460 and HCC827 cells were cultured in the presence or absence of a PRMT5-inhibiting molecule (GSK591) at the indicated concentrations for 4 days. Western blot analysis of NCI-H460 cells (left) and HCC827 cells (right) (symmetric dimethylated arginine sDMR and PRMT5 expression; β-actin served as loading control. One of three similar results is shown. **(B)** NCI-H460 and HCC827 cells were cultured in the presence or absence of 250 nM GSK591, and PD-L1 expression was measured by Western blot analysis (top) and CD274 expression by qPCR (bottom) after 4 days. Data are the means ± SEM of three independent experiments. Statistical differences were determined by a two-tailed unpaired Student’s t test, ***p < 0.001, ****p < 0.0001. **(C)** NCI-H460 and HCC827 cells were cultured in the presence or absence of 250 nM GSK591, and PD-L1 expression was analyzed by flow cytometry on days 0, 2, 4, and 6. Data represent the mean fluorescence intensity (MFI). One of three similar results is shown. **(D, E)** LLC cells were treated in the presence or absence of GSK591 at the indicated concentrations; PD-L1 expression was determined on days 2, 4, and 6 by flow cytometry **(D)** and qPCR **(E)**. Data represent the mean fluorescence intensity (MFI) and *Cd274* mRNA normalized to *Actb.* **(F, G)** Nude mice bearing LLC tumors were treated with GSK591 (50 mg/kg) or vehicle (n = 5/group) for 12 days. PD-L1 protein expression was measured by histochemistry **(F)**, and *Cd274* mRNA expression was measured by qPCR **(G)**. Representative HE and IHC images for PD-L1 expression of the tumor sections from the vehicle and GSK591-treated groups are shown. Scale bar = 100 μm. Means ± SEM are plotted. *p < 0.05, **p < 0.01, *** *p* < 0.001, ****p < 0.0001.

We evaluated the effect of a PRMT5-specific inhibitor in human and mouse lung cancer cell lines. First, we determined the appropriate concentration of GSK591, which has been reported as a PRMT5 inhibitor (20). We treated the lung cancer cell lines, HCC827 and NCI-H460, with various concentrations of GSK591 for 4 days. Symmetric pan-dimethyl arginine (SDMR) expression was markedly decreased following treatment of lung cancer cell lines with 250 nM GSK591 (**Figure 2A**). Next, we treated HCC827 and NCI-H460 with GSK591 at 250 nM separately and analyzed PD-L1 mRNA and protein expression. Consistent with the PRMT5 shRNA experiments, the PRMT5 inhibitor increased *CD274* and PD-L1 expression (**Figure 2B**). Moreover, when the cells were treated with GSK591 at different time points and analyzed by flow cytometry, PD-L1 expression was significantly increased in a time-dependent manner in both HCC827 and NCI-H460 cells (**Figure 2C**). In addition, we treated LLC with GSK591 and evaluated *CD274* mRNA and PD-L1 protein levels. Similar to the results with human lung cancer cell lines, PD-L1 mRNA and protein expressions were increased at 1 μM in a time-dependent manner (**Figures 2D, E**).

To further evaluate the *in vivo* effects of PRMT5 inhibition on *CD274* expression, we performed an *in vivo* study in which LLC cells were implanted subcutaneously into nude mice and treated with 50 mg/kg GSK591 once a day. After 12 days of continuous treatment, animals were euthanized, and the tumors were resected. We found that GSK591 treatment significantly reduced tumor weight and volume compared with vehicle treatment (**Supplementary Figures 2A, C**). Also, SDMR expression was decreased in the GSK591-treated group (**Supplementary Figure 2D**). We then used qPCR, Western blot analysis, and immunohistochemistry (IHC) to evaluate PD-L1 expression in tumor tissues. Interestingly, both *CD274* mRNA and PD-L1 protein expression increased in tumor tissue from the GSK591-treated group (**Figures 2F, G**, **Supplementary Figure 2E**). Collectively, our *in vitro* and *in vivo* studies confirmed that PRMT5 inhibition induced PD-L1 expression.

### PRMT5 Regulates PD-L1 Expression Through Symmetric Dimethylation of Histone H4R3

**Figure 3:**
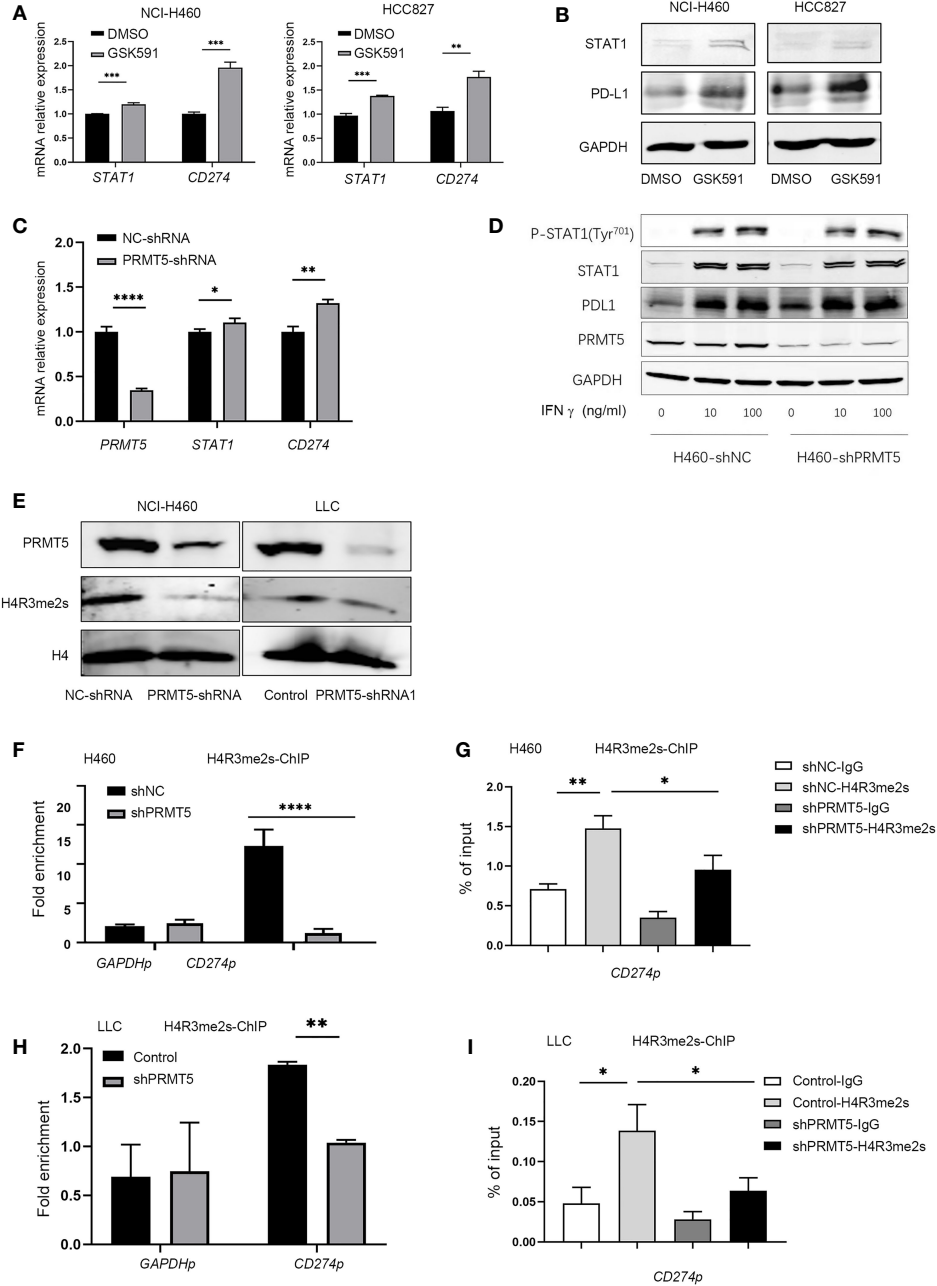
Inhibition of PRMT5 upregulates PD-L1 expression through symmetric dimethylation of H4R3. **(A, B)** NCI-H460 and HCC827 cells were cultured in the presence or absence of 250 nM GSK591 for 4 days, and *STAT1* and *CD274* transcripts were measured by qPCR **(A)**. Data are the means ± SEM of three independent experiments. Statistical differences were determined by a two-tailed unpaired Student’s t test, **p < 0.01, ***p < 0.001. STAT1 and PD-L1 expressions were detected by Western blot analysis and GAPDH served as a loading control **(B)**; one of three similar results are shown. **(C)** PRMT5 was knocked down in NCI-H460 cells, and *STAT1* and *CD274* mRNA expression was determined by qPCR. Data are the means ± SEM of three independent experiments. Statistical differences were determined by two-tailed unpaired Student’s t test, *p < 0.05, **p < 0.01, ***p < 0.001. **(D)** NC-shRNA- and PRMT5-shRNA-treated NCI-H460 cells were stimulated with recombinant human IFNγ at the indicated concentrations for 12 h. Total and phosphorylated STAT1, PD-L1, and PRMT5 were analyzed by Western blot analysis. GAPDH served as a loading control. **(E)** Immunoblot of H4R3 me2s in NCI-H460 and LLC cells after PRMT5 was knocked down; H4 served as a loading control. One of three similar results is shown. **(F, G)** PRMT5 was knocked down in NCI-H460 cells, and ChIP qPCR was performed to determine H3R2 me2s binding to the *CD274* promoter locus (*CD274*p) in NCI-H460 shPRMT5 cells compared with shNC cells. IgG or anti-H4R3 me2s antibodies were utilized in the chromatin immunoprecipitation assay. Fold enrichment was calculated as the H4R3 me2s signal/IgG signal for each sample **(F)**. The *GAPDH* promoter locus (*GAPDH*p) was utilized as a negative control. qRT-PCR values from IgG and H4R3 me2s were normalized by the input qRT-PCR values **(G)**. Data are the means ± SEM of three independent experiments. Statistical differences were determined by a two-tailed unpaired Student’s t test, *p < 0.05, **p < 0.01, ***p < 0.001, ****p < 0.0001. **(H, I)** PRMT5 was knocked down in LLC cells, and ChIP qPCR was performed to determine H4R3 me2s binding to the *CD274* promoter locus (*CD274*p) in LLC shPRMT5 cells compared with control cells. IgG or anti-H4R3 me2s antibodies were utilized in the chromatin immunoprecipitation assay. Fold enrichment was calculated as the H4R3 me2s signal/IgG signal for each sample **(H)**. The *GAPDH* promoter locus (*GAPDH*p) was utilized as a negative control. qRT-PCR values from IgG and H4R3 me2s were normalized by the input qRT-PCR values **(I)**. Data are the means ± SEM of three independent experiments. Statistical differences were determined by a two-tailed unpaired Student’s t test, *p < 0.05, **p < 0.01.

We explored the mechanisms of PD-L1 regulation by PRMT5. Previous reports indicated that IFN-γ upregulates PD-L1 expression through the JAK/STAT-1 pathway (21). Therefore, we used qPCR and Western blot analysis to measure the expression of STAT1 and PD-L1 after PRMT5 inhibition. The results indicated increased the expressions of STAT1 and PD-L1 mRNA; however, STAT1 increased to a lesser degree compared with PD-L1 (**Figure 3A**). STAT1 protein expression levels in the PRMT5 inhibition group were slightly increased (**Figure 3B**). We further confirmed these findings in shRNA knockdown experiments, and the results were consistent with PRMT5 inhibition by GSK591 (**Figure 3C**). Moreover, when cells were stimulated with IFNγ, total STAT1 and p-STAT1 expression levels were increased, but there was no significant difference between the PRMT5 shRNA and control groups (**Figure 3D**). Our data indicated that PRMT5 regulated PD-L1 expression primarily through IFNγ-STAT1-dependent pathways; however, other mechanisms may be involved in the regulation of these pathways. PRMT5 is a type II arginine methyltransferase and has been reported to play an important role in histone and non-histone methylation during cell signaling, which further attenuates the transcription factor expression. One mechanism by which PRMT5 suppresses gene expression is through the methylation of H4R3 (H4R3me2s), which contributes to transcriptional repression (22). We measured H4R3me2s expression in PRMT5 shRNA NCI-H460 cells, and the results showed that knockdown of PRMT5 decreased H4R3me2s expression (**Figure 3E**). Furthermore, a ChIP PCR assay was performed to verify whether H4R3me2s repressed *PD-L1* transcription. The results showed that H4R3me2s binding to the *CD274* promoter was decreased when PRMT5 was knocked down in NCI-H460 cells (**Figures 3F, G**). We also verified ChIP PCR in LLC cells. The data were consistent with that of the human lung cancer cell line. H4R3me2s binding to the murine *CD274* promoter was reduced in the PRMT5 shRNA group compared with the control group (**Figures 3H, I**). Collectively, these findings suggest that PRMT5 regulates PD-L1 expression *via* PRMT5-mediated epigenetic repression.

### PRMT5 Inhibition Reduces CD8 T Cells Effector Function *In Vitro*

**Figure 4:**
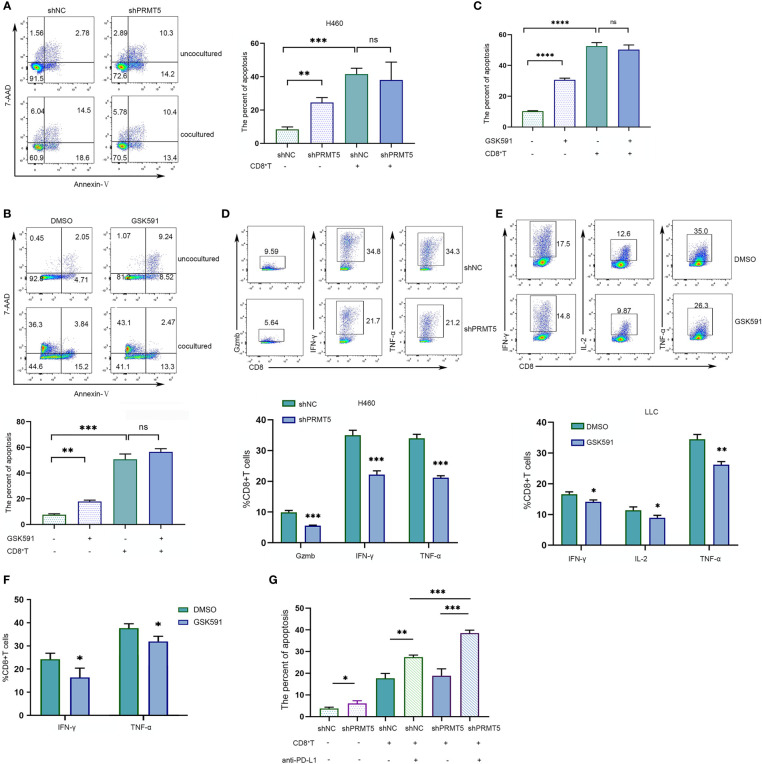
PRMT5 inhibition reduces CD8 T cell antitumor activity by inducing PD-L1 expression in lung cancer lines. **(A)** NC-shRNA and PRMT5-shRNA NCI-H460 cells cultured alone or cocultured with human CD8 T cells; NCI-460 apoptosis was analyzed by flow cytometry. Representative plots are shown (left). Bar graph (right) shows the means ± SEM of three independent experiments. Statistical differences were determined by a two-tailed unpaired Student’s t test, **p < 0.01, ***p < 0.001, ns, not significant. **(B)** LLC cells were treated with 250 nM GSK591 and cultured alone or with mouse CD8 T cells. LLC apoptosis was analyzed by flow cytometry. The representative plots (upper) are shown; bar graph (bottom) indicates the means ± SEM of three independent experiments. Statistical differences were determined by a two-tailed unpaired Student’s t test, **p < 0.01, ***p < 0.001. **(C)** The LLC cell line was pretreated with GSK591 at 250 nM for 4 days and washed thoroughly with culture medium, then cocultured with CD8 T cells for another 24 h. Data show the percent apoptosis of the LLC cells. Bar graph shows the means ± SEM of three independent experiments. Statistical differences were determined by a two-tailed unpaired Student’s t test, ****p < 0.0001, ns—no significant. **(D)** CD8 T cells were cocultured with NC-shRNA and PRMT5-shRNA NCI-H460 cells for 48 h. Gzmb; IFN-γ; TNF-α expressions were assessed by flow cytometry. Bar graph (bottom) shows the means ± SEM of three independent experiments. Statistical differences were determined by a two-tailed unpaired Student’s t test, ***p < 0.001. **(E)** CD8^+^ T cells were cocultured with DMSO-treated and GSK591-treated LLC cells. IL-2, IFN-γ, and TNF-α expressions were determined by flow cytometry. Bar graph (bottom) shows the means ± SEM of three independent experiments. Statistical differences were determined by a two-tailed unpaired Student’s t test, *p < 0.05, **p < 0.01. **(F)** The LLC cell line was pretreated with GSK591 at 250 nM for 4 days and washed thoroughly with culture medium, then cocultured with CD8 T cells for another 24 h. IFN-γ and TNF-α expressions were determined by flow cytometry. Bar graph shows the means ± SEM of three independent experiments. Statistical differences were determined a by two-tailed unpaired Student’s t test, *p < 0.05. **(G)** CD8 T cells were cultured with NC-shRNA and PRMT5-shRNA NCI-H460 cells, and anti-PD-L1 or control IgG was added. NCI-H460 apoptotic cells were determined by flow cytometry. Bar graph shows the means ± SEM of three independent experiments. Statistical differences were determined by a two-tailed unpaired Student’s t test, *p < 0.05, **p < 0.01, ***p < 0.001.

Previously, we found that PRMT5 inhibition by PRMT5 shRNA or a selective chemical inhibitor induced *CD274* mRNA and PD-L1 protein expression. We hypothesized that PRMT5 inhibition may cause immune-resistance in addition to direct antitumor effects in lung cancer. Therefore, PRMT5 shRNA or control shRNA NCI-H460 stable transformation cells were cocultured with CD8 T cells to evaluate the CD8 T cell antitumor activity after PRMT5 expression was inhibited. The data showed that knockdown of PRMT5 in NCI-H460 cells decreased survival compared with the control group, suggesting that PRMT5 directly affects lung cancer line survival. However, when these cells were cocultured with human CD8 T cells and stimulated with anti-CD3 and CD28 antibodies, while apoptosis of NCI-H460 cells was increased compared with cultured cells alone, there was no difference between the PRMT5 shRNA and control groups in the coculture system (**Figure 4A**). Notably, HLA-mismatched PBMC-derived CD8 T cells and NCI-H460 may react in an allogeneic fashion in this coculture system. Next, we used mouse CD8 T cells and cocultured them with LLC cells. Following GSK591 treatment, the tumor-killing ability was reduced compared with the control group (**Figure 4B**). These results suggest that PRMT5 inhibitors have negative effects on T cell activation and cytokine secretion (23), so we pretreated LLC cells with GSK591 for 4 days, washed them thoroughly with culture medium, cocultured them with CD8 T cells for another 24 h, and measured CD8 T cell antitumor activity. The results indicated that the antitumor ability of CD8 T cells was decreased in GSK591-pretreated LLC cells compared with the control (**Figure 4C**). In addition, we measured CD8 T cell inflammatory cytokine expression in the cocultured system. The results indicated that CD8 T cells, when cocultured with PRMT5 shRNA knockdown stable cell lines, granzyme B, IFNγ, and TNFα expressions were decreased compared with the control group (**Figure 4D**). For LLC cells cocultured with murine CD8 T cells in the presence of GSK591, IFNγ, IL-2, and TNFα expression was reduced compared with the DMSO group (**Figure 4E**). Similarly, GSK591-pretreated LLC cells reduced murine CD8 T cell expression of IFNγ and TNFα in the coculture system (**Figure 4F**). These data suggest that PRMT5 inhibition directly affected tumor cells but also reduced the antitumor activity of CD8 T cells, which may result from a higher expression of PD-L1 when tumor cell PRMT5 expression was inhibited. As expected, when the PD-L1-specific antibody was added into the coculture system, the CD8 T cell antitumor activity was restored (**Figure 4G**). Thus, our *in vitro* data support a link between PRMT5 inhibition and immune-resistance mediated by PD-L1 expression.

### Combined PRMT5 Inhibition and Anti-PD-L1 Antibody Enhances Antitumor Ability

**Figure 5:**
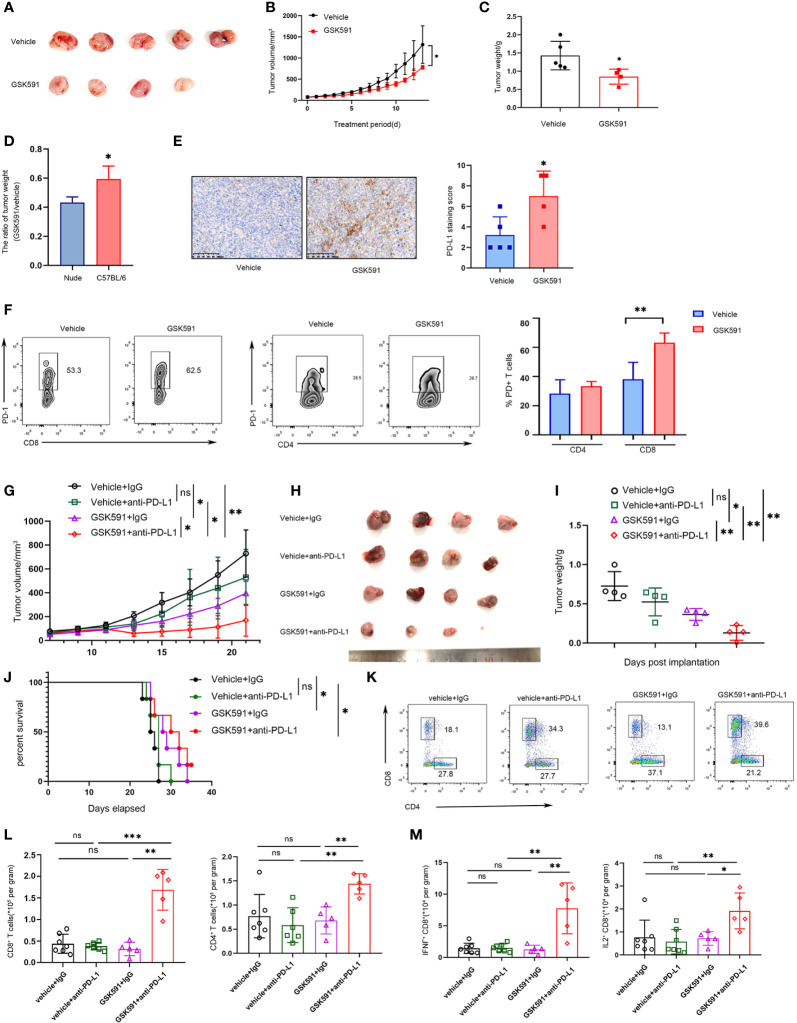
Combined PRMT5 inhibition and anti-PD-L1 antibody enhances antitumor activity. **(A–D)** C57BL/6 mice bearing LLC tumors were treated with GSK591 (50 mg/kg) or vehicle (n = 5/group) for 12 days. Images of resected tumors **(A)**, tumor growth curves **(B)**, and tumor weights **(C)** are shown. The tumor weight ratio of GSK591 treated with vehicle in C57BL/6 mice and nude mice **(D)**. One of three similar results is shown. Bar graph shows the means ± SEM of three independent experiments. Statistical differences were determined by a two-tailed unpaired Student’s t test, *p < 0.05. **(E)** PD-L1 expression was determined by IHC in GSK591 or the vehicle-treated group. Statistical differences were determined by a two-tailed unpaired Student’s t test, **p < 0.01. **(F)** PD1 expression was determined in infiltrated CD8T and CD4T cells by flow cytometry. Statistical differences were determined by a two-tailed unpaired Student’s t test, **p < 0.01. **(G–J)** C57BL/6 mice bearing LLC tumors were treated with IgG (10 mg/kg), anti-PD-L1 (10 mg/kg), and GSK591 (50 mg/kg) or the combination (n = 4 per group). Tumor volumes **(G)**, images of resected tumor tissues **(H)**, tumor weights **(I)**, and survival of mice **(J)** are shown. Statistical differences were determined by a two-tailed unpaired Student’s t test, *p < 0.05, *p < 0.01. For the survival curve, the *p* value was established by the Mantel–Cox test. **(K, L)** Flow cytometry analysis of tumor infiltrating CD8 T and CD4T cells after anti-PD-L1 and GSK591 treatment. Representative plots are shown **(K)**. Bar graph shows the means ± SEM **(L)**. Statistical differences were determined by a two-tailed unpaired Student’s t test, **p < 0.01, ***p < 0.001, ns—no significant. **(M)** IFNγ and IL-2 expressions in tumor-infiltrating CD8 T cells were analyzed by flow cytometry. Bar graph shows the means ± SEM. Statistical differences were determined by a two-tailed unpaired Student’s t test, *p < 0.05, **p < 0.01, ns, not significant.

Based on previous findings, we demonstrated that blocking PRMT5 expression has an antitumor effect, but it also induces PD-L1 expression. Thus, PMRT5 inhibition alone may not be the optimal therapy for lung cancer. We hypothesize that combining the anti-PD-L1 antibody with the PRMT5 inhibitor may achieve a better effect than either therapy alone. Indeed, when C57BL/6 LLC-bearing mice were treated with GSK591 (50 mg/kg, od) alone, tumor weight and volume were deceased compared with the vehicle; however, the effect was less than that observed for the nude mice (**Figures 5A–D**). Moreover, PD-L1 expression was increased in the GSK591-treated group compared with the control group (**Figure 5E**). In addition, the proportion of PD1-expressing CD8 T cells was increased in the GSK591-treated group, but the percentage of PD-1+ CD4 cells was unchanged (**Figure 5F**). Moreover, tumor-infiltrating CD4 and CD8 T cells were decreased in the GSK591-treated group, whereas infiltrating NK, B, macrophages, and Treg cells were not significantly different between the two groups (**Supplementary Figures 3A-E**). These data suggest that GSK591 treatment influences T cell function. Next, we treated mice with a combination of GSK591 and anti-PD-L1. C57BL/6 mice treated with anti-PD-L1 alone exhibited no significant antitumor response in the LLC mouse model (**Figures 5G–I**). However, marked tumor regression was observed in the combination group compared with GSK591 alone (**Figures 5G–I**). Although the combination therapy did not prolong survival (**Figure 5J**), tumor-infiltrated CD8 T and CD4 T cells were significantly increased in the combination groups (**Figures 5K, L**). Combination treatment resulted in increased IFNγ and IL-2 secretion by CD8 but not CD4 T cells (**Figure 5M** and **Supplementary Figure 4**). Our data suggest that combined PRMT5 inhibition and anti-PD-L1 antibody therapy influences CD8 T cell function and achieved better effects compared with single-agent therapy.

## Discussion

Anticancer therapies that target tumor-specific genetic represent a promising strategy; however, drugs might also impair tumor-activated immune cells while targeting the highly proliferative cancer cells. PRMT5 is the main type II enzyme that catalyzes symmetric dimethylarginine of histone proteins to induce gene silencing by producing repressive histone marks, such as H2AR3 me2s, H3R8 me2s, and H4R3 me2s (3, 24, 25). PRMT5 overexpression has been found to occur in various malignancies. Moreover, PRMT5 exhibits pleiotropic effects in other cell types, such as hematopoietic stem cells and T cells. Thus, PRMT5 inhibitors may have cell type-specific effects on gene expression and growth. Targeting PRMT5 in cancer patients with inhibitors may also result in other side effects, such as myelosuppression. The clinical activity and side effects of PRMT5 inhibitors need to be defined *in vivo* to obtain the best treatment response. Our findings indicate that inhibition of PRMT5 by shRNA or a pharmacological approach attenuated tumor progression; however, they also induced cancer cell interferon-related gene and PD-L1 expression. Given the importance of T cells to the tumor microenvironment, we hypothesized that PRMT5 inhibition may have negative immunomodulatory effects on T cells through the PD-L1/PD1 axis. Indeed, when PRMT5 was knocked down in lung cancer lines and cocultured with CD8 T cells *in vitro*, we found that the CD8 T cells exhibited reduced antitumor activity compared with control cells. Furthermore, targeting PRMT5 in immunocompromised mice with lung cancer resulted in reduced tumor progression; however, the treatment effect was reduced significantly in immunocompetent mice and associated with increased PD-L1 expression in the tumor tissue. Moreover, PRMT5 inhibition induced PD1 expression, which mitigates CD8 T cell antitumor activity. Mechanistically, we found that tumors lacking PRMT5 showed decreased H4R3 me2s deposition on the *STAT1* and *CD274* promoters and exhibited increased expression of genes for both type I IFN and the immune checkpoint ligand, *CD274*.

The PRMT5 inhibitors, EPZ015666 and DST-437, suppress Tregs and CD8T cells. EPZ015666 directly upregulates p53 and impairs the AKT signaling pathway in human CD8 T cells (16, 26). Interestingly, PRMT5 depletion antagonizes melanoma growth in immunocompetent, but not immunocompromised, mice. The mechanism is that PRMT5 inhibition increases the abundance of infiltrated immune cells, but has no obvious effects on melanoma cells (27). Monotherapy is ineffective; combination of either genetic or pharmacological PRMT5 inhibitors with anti-PD-1 therapy results in the inhibition of melanoma growth, which is CD8 T cell-dependent (27). However, we demonstrated that targeting PRMT5 induced PD-L1 expression in cancer cells, and it compromised CD8 T cell antitumor immune responses in immunocompetent mice, suggesting that PRMT5 inhibition may have different effects on the immune cell functions in different tumor types. We further established the molecular mechanisms through which inhibition of PRMT5 activity indirectly compromised the antitumor response by T cells through histone methylation, providing a compelling new pathway to target. We also provided strong evidence for the essential partnership between PRMT5 inhibition and anti-PD-L1 therapy in lung cancer to improve treatment efficacy. Our findings emphasize the importance of considering side effects on the immune system when developing strategies to inhibit PRMT5 and provide a rationale for combination therapy with anti-PD-L1 therapy in lung tumors treated with PRMT5 inhibitors.

Currently, GSK3326595, JNJ-64619178, and PF-06939999, three PRMT5 inhibitors with different mechanisms of action against PRMT5 and its S-adenosylmethionine substrate, are being evaluated in four clinical trials for both hematologic and solid tumors (Clinical Trial Nos.: NCT03614728, NCT02783300, NCT03573310, NCT03854227). PRMT5 depletion is known to be embryonically lethal; thus, treatment with PRMT5 inhibitors, such as GSK3326595, may induce toxicity later in clinical trials. To date, little is known about how PRMT5 inhibitors affect immune cells in the tumor microenvironment or the human immune system. In summary, our study revealed that PRMT5 inhibitors represent a double-edged sword as they can selectively kill cancer cells but may also deter the antitumor immune response. We show that combination PRMT5/PD-L1 inhibition may achieve a better antitumor effect: targeting PRMT5 to directly inhibit tumor cell survival and targeting PD-L1 to overcome the side effects of targeting PRMT5 and enhance immune function. Overall, our findings address an unmet clinical need through the combination of PRMT5 inhibition and anti-PD-L1 therapy, which may represent a promising strategy for lung cancer therapy.

## Data Availability Statement

The original contributions presented in the study are publicly available. These data can be found here: https://www.ncbi.nlm.nih.gov/geo/, accession: GSE178521.

## Ethics Statement

The studies involving human participants were reviewed and approved by the Research Ethics Board of Xinhua Hospital, Shanghai Jiao Tong University School of Medicine. The patients/participants provided their written informed consent to participate in this study. The animal study was reviewed and approved by the Institutional Animal Care and Use guidelines, Xin Hua Hospital Committee.

## Author Contributions

LS, YZ, and HX designed the experiments. BZ, RH, ZC, SC, and NC, performed the experiments. YZ and BZ analyzed the data. LS, YZ, and BZ wrote the manuscript. All authors contributed to the writing and providing of feedback. All authors contributed to the article and approved the submitted version.

## Funding

This work was supported by grants from the National Natural Science Foundation of China [81571525, 81873863, and 82071753 to YZ], the Shanghai Municipal Education Commission—Gaofeng Clinical Medicine Grant Support [20161315 to YZ], the Key Specialty Development Program of Xin Hua Hospital and Shanghai Municipal Health Commission (to LS), and Medicine and Engineering Cross Research Foundation of Shanghai Jiao Tong University (Project No. YG2017ZD02 to LS).

## Conflict of Interest

The authors declare that the research was conducted in the absence of any commercial or financial relationships that could be construed as a potential conflict of interest.

## Publisher’s Note

All claims expressed in this article are solely those of the authors and do not necessarily represent those of their affiliated organizations, or those of the publisher, the editors and the reviewers. Any product that may be evaluated in this article, or claim that may be made by its manufacturer, is not guaranteed or endorsed by the publisher.
