# Supplementary material for: PRMT5 Inhibition Promotes PD-L1 Expression and Immuno-Resistance in Lung Cancer
Source: Front Immunol. 2022 Jan 17;12:722188. doi: 10.3389/fimmu.2021.722188 (PMC8801487; doi:10.3389/fimmu.2021.722188)
Supplement: Supplementary file 1 [file DataSheet_1.pdf]

# PRMT5 inhibition promotes PD-L1 expression and immuno-resistance in lung cancer

Rui Hu[1†], Bingqian Zhou[2†], Zheyi Chen[2], Shiyu Chen[2], Ningdai Chen[2], Lisong Shen[2, 3, 4*], Haibo Xiao[1*],Yingxia Zheng[2*]

[1] Department of Thoracic Surgery, Xin Hua Hospital, Shanghai Jiao Tong University School of Medicine, Shanghai, China; [2] Department of Laboratory Medicine, Xin Hua Hospital, Shanghai Jiao Tong University School of Medicine, Shanghai, China; [3]Faculty of Medical Laboratory Science, Shanghai Jiao Tong University School of Medicine, Shanghai, China. [4]Xin Hua Children's Hospital, Shanghai Jiao Tong University School of Medicine.

† These authors contributed equally to this paper.

* Correspondence e-mails: lisongshen@hotmail.com (L.S.); xiaohaibo@xinhuamed.com.cn (H.X.); zhengyingxia@xinhuamed.com.cn (Y.Z.).

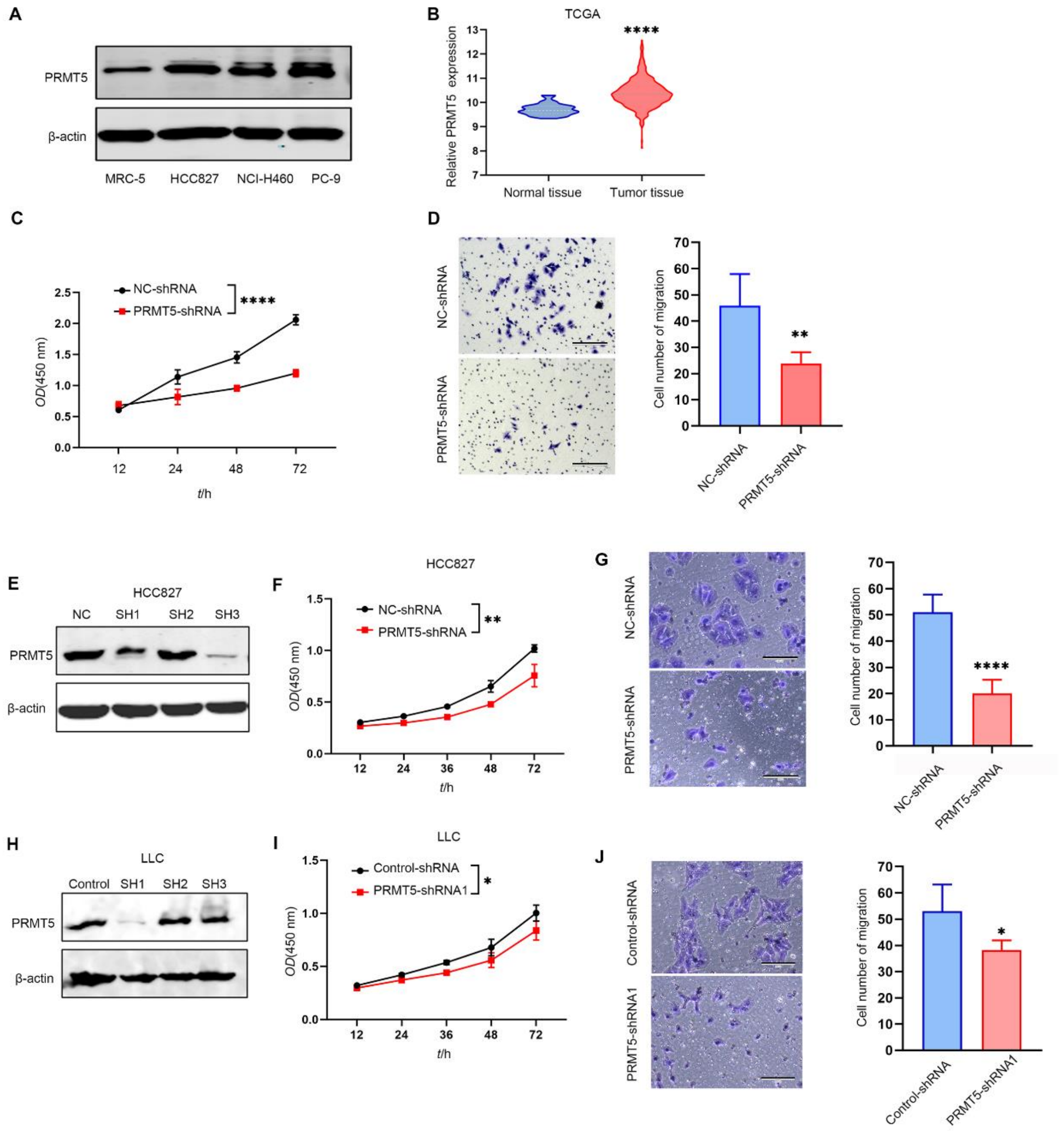


**Supplementary Figure.1 PRMT5 expression in Lung cancer cell and its effects on proliferation and migration of lung cancer cell lines.**

(**A**) PRMT5 expression in human embryonic lung fibroblasts and lung cancer cells lines was detected by western blot, β-actin as the loading control. (B) Analysis of TCGA RNA-seq data of human lung adenocarcinoma, violin plots showed the relative *PRMT5* expression between normal tissues and tumor tissues. Statistical differences were determined by two-tailed unpaired Student's t test, ****$P < 0.0001$. (**E,H**) Three sites of shRNA sequence targeting human PRMT5 or murine PRMT5 were designed, separately. *PRMT5* specific or control shRNA were transfected into human lung cancer line HCC827(**E**) and murine lung cancer cell line LLC(**H**).PRMT5 expression was measured by western blotting, β-actin as the loading control. (**C, F and I**)Effects of *PRMT5*-shRNA on the proliferation of NCI-H460(**C**), HCC827 (**F**) and LLC (**I**) cells were detected by CCK8 assay. Statistical differences were determined by two-tailed unpaired Student's t test, ****$P < 0.0001$. (**D, G, J**) Effects of *PRMT5*-shPRMT5 on the migration ability of NCI-H460 (**D**), HCC827 (**G**) and LLC

(**J**) cells. Scale bar 200μm. Data were the means ± SEM of three independent experiments. Statistical differences were determined by two-tailed unpaired Student's t test, $**P < 0.01$.

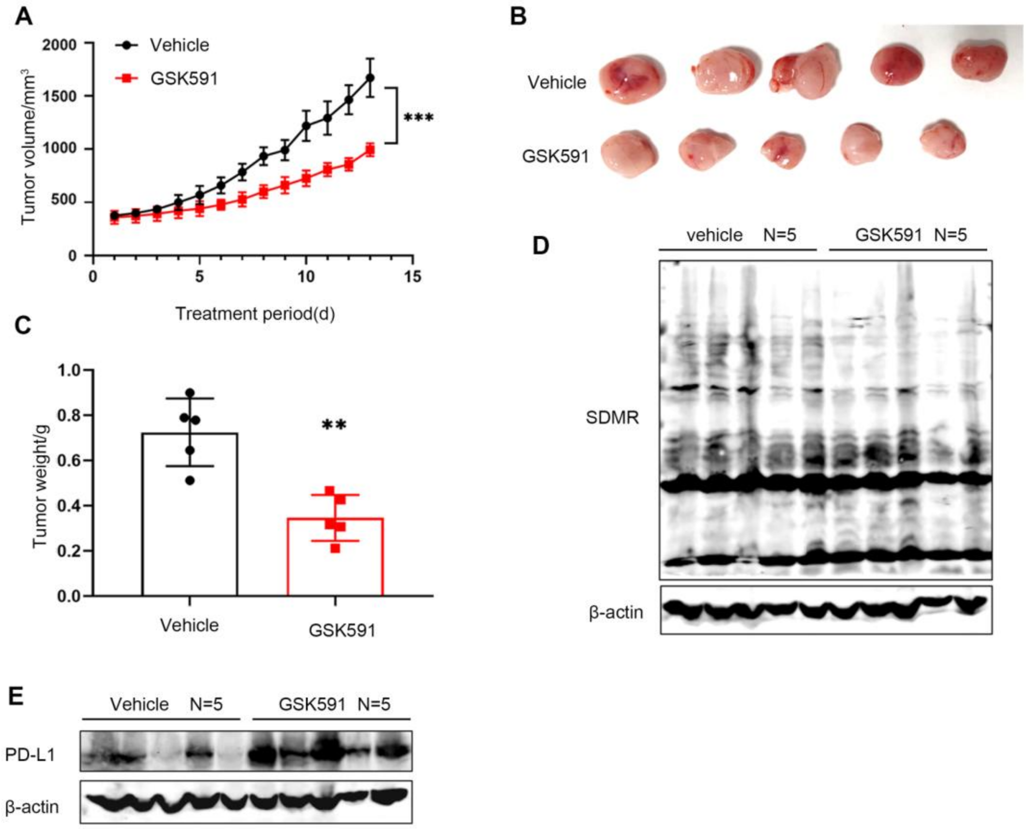


**Supplementary Figure 2 Inhibition of PRMT5 suppresses lung cancer cell growth in nude mice.**

(**A, B and C**) Nude mice bearing LLC tumors were treated with GSK591 (50mg/kg) or vehicle (n=5/group) for 12 days. Tumor growth curves (**A**), images of resected tumor (**B**), and tumor weights (**C**) were shown. Statistical differences were determined by two-tailed unpaired Student's t test, $**P < 0.01$, $***P < 0.001$. (**D and E**) SDMR (**D**) and PD-L1 (**E**) expression was analysis of tumor sections (N=5 each group) from vehicle and GSK591 treated group by western blot; β-actin served as loading control.

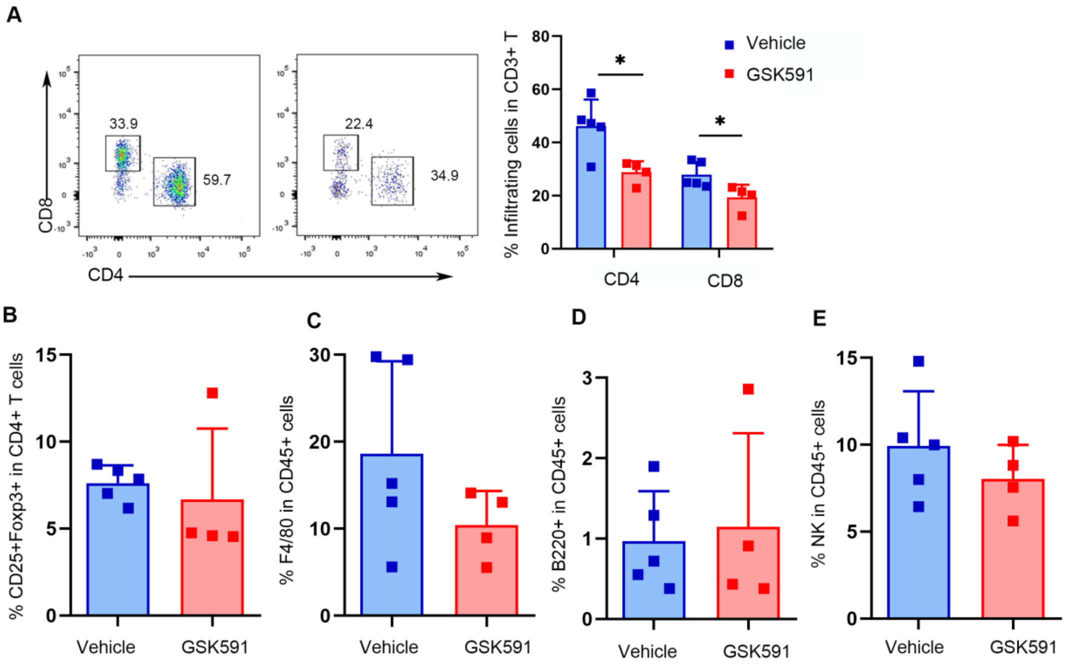


**Supplementary Figure 3 RMT5 inhibition reduced T cell infiltration into lung tissue.**

C57BL/6 mice bearing LLC tumor were treated with GSK591 (50mg/kg, n=4) or vehicle (n=5) for 12 days. Mice were sacrificed on day 20 after implantation. (**A**) Flow cytometry analysis of tumor infiltrating $CD8^{+}$ T and $CD4^{+}$ T cells. The representative plots were shown (Left). Bar graph was the means ± SEM (Right). Statistical differences were determined by two-tailed unpaired Student's t test, $^{*}P < 0.05$.(**B-E**) Flow cytometry analysis of tumor infiltrating Treg cells (**B**), macrophages (**C**), B cells (**D**) and NK cell (**E**).

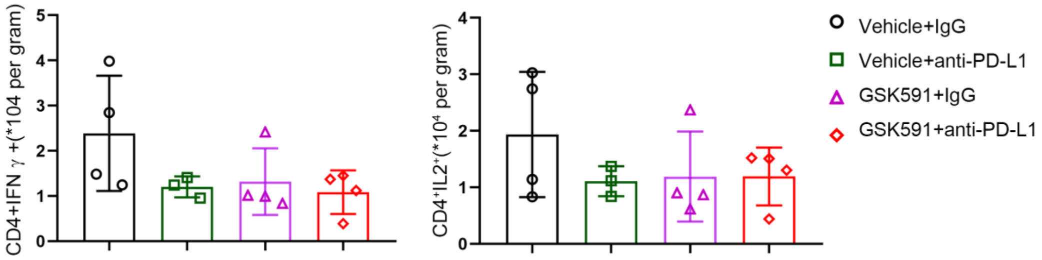


**Supplementary Figure 4 Combined PRMT5 inhibition and anti-PD-L1 antibody treatment had no impact on CD4 T cells cytokines expression.**
C57BL/6 mice bearing LLC tumors following treatment with IgG (10mg/kg), anti-PD-L1 (10mg/kg), GSK591 (50mg/kg) and the combination (n=4 per group) (day 18 post tumor cell implantation). Quantification of CD4 T cells expressing effector markers (IFNγ and IL-2) as per gram of tumor.

**Supplementary Table 1. List of antibodies used for flow cytometry**

| antibody | Company | Cat# |
|---|---|---|
| PE Anti-mouse PDL1 | Biolegend | 124307 |
| BV421 Anti-human PDL1 | BD | 563738 |
| APC-cy7 Anti-human CD8 | Biolegend | 344714 |
| Percpcy5.5 Anti-mouse CD8 | BD | |
| FITC Anti-Mouse CD4 | BD | 557307 |
| Annexin V PE Apop Dtec Kit | BD | 559763 |
| BV421 Anti-human IFNγ | BD | 562988 |
| APC Anti-human TNF-α | Invitrogen | 17-7349-82 |
| PE Anti-human Granzyme B | Biolegend | 372208 |
| BV421 Anti-mouse TNFα | Invitrogen | 48-7321-82 |
| APC Anti-mouse IFNγ | Invitrogen | 17-7311-81 |
| PE-Cy™7 Anti-Mouse CD45 | BD | 552848 |
| APC-cy7 Anti-mouse CD3 | BD | 557596 |
| PerCP/Cyanine5.5 Anti-human CD4 | Biolegend | 317428 |
| BV421 Anti-mouse CD8a | Biolegend | 100737 |
| PE Anti-mouse IL-2 | Invitrogen | 12-7021-82 |

**Supplementary Table 2. Primers sequences**

| Genes | Species | Sequence(5’-3’) |
|---|---|---|
| *Prmt5*-F | Mouse | CTGAATTGCGTCCCCGAAATA |
| *Prmt5*-R | Mouse | AGGTTCCTGAATGAACTCCCT |
| *Actb* -F:， | Mouse | TGTCCACCTTCCAGCAGATGT |
| *Actb* -R: | Mouse | AGCTCAGTAACAGTCCGCCTAG |
| *CD274*-F | Human | ACAGCTGAATTGGTCATCCC |
| *CD274*-R | Human | TGTCAGTGCTACACCAAGGC |
| *GAPDH*-F | Human | GGAGCGAGATCCCTCCAAAAT |
| *GAPDH*-R | Human | GGCTGTTGTCATACTTCTCATGG |
| *STAT1*-F | Human | CGGCTGAATTTCGGCACCT |
| *STAT1*-R | Human | CAGTAACGATGAGAGGACCCT |
| *Cd274*-F | Mouse | AAGCCTCAGCACAGCAACTTCAG |
| *Cd274*-R | Mouse | TGTAGTCCGCACCACCGTAGC |
| *CD274*-Promoter-F | Human | AACCAATGCAAGGGCTATCTC |
| *CD274*-Promoter-R | Human | GTGCCTGTGTGCTCCCTTTTC |
| *GAPDH*-Promoter-F | Human | CTGAGCAGACCGGTGTCACATC |
| *GAPDH*- Promoter-R | Human | GAGGACTTTGGGAACGACTGAG |
| *Gapdh*-Promoter-F | Mouse | CTCAGGGCGCGAAAGTAAAG |
| *Gapdh*-Promoter-R | Mouse | CGGCCCGGAGTCTTAAGTAT |
| *Cd274*-Promoter-F | Mouse | CACCCCTGCTTTCACTGTTG |
| *Cd274*-Promoter-R | Mouse | GTGAAGTTTCCGCAGACCAC |
